# Defining “High-In” Saturated Fat, Sugar, and Sodium to Help Inform Front-of-Pack Labeling Efforts for Packaged Foods and Beverages in the United States

**DOI:** 10.3390/nu16244345

**Published:** 2024-12-17

**Authors:** Elizabeth K. Dunford, Donna R. Miles, Bridget A. Hollingsworth, Samantha Heller, Barry M. Popkin, Shu Wen Ng, Lindsey Smith Taillie

**Affiliations:** 1 The George Institute for Global Health, University of New South Wales, Sydney, NSW 2000, Australia; edunford@georgeinstitute.org.au; 2 Carolina Population Center, University of North Carolina, Chapel Hill, NC 27516, USA; drmiles@email.unc.edu (D.R.M.); bridget@unc.edu (B.A.H.); sph@unc.edu (S.H.); popkin@unc.edu (B.M.P.); shuwen@unc.edu (S.W.N.); 3 Department of Nutrition, Gillings School of Global Public Health, University of North Carolina, Chapel Hill, NC 27516, USA

**Keywords:** US FDA, HFSS, front-of-pack labeling, processed foods, nutrient profiling

## Abstract

**Background**: To help consumers make healthier choices, the US Food and Drug Administration (FDA) has been charged with developing a front-of-package label (FOPL) to appear on US packaged foods and beverages. One option being explored is the use of “high-in” FOPLs for added sugar, sodium, and saturated fat using a threshold of ≥20% of the recommended daily value (%DV) per portion/serving size to define “high-in”. While research has addressed what FOPL designs are most effective at visually communicating “high-in”, less attention has been paid to the nutrient profile model (NPM) used to decide which products should receive these labels. In addition, several established regional NPMs already exist that identify products that are high in nutrients of concern, but it is unclear how these compare to the FDA’s %DV approach. **Methods**: We used a dataset of 51,809 US products from Mintel’s Global New Products Database to examine how the FDA’s current definition of “high-in” compares to three established regional NPMs: the Canadian NPM, the Pan American Health Organization (PAHO) NPM, and Chile’s NPM. **Results**: Overall agreement between the four NPMs was 51% for foods and 72% for beverages, with highest agreement in categories such as sweetened sodas (87%), and lowest agreement in categories such as bread (14%) and salty snacks (29%). The Canadian NPM showed the highest agreement to the FDA “high-in” criteria while the Chilean and PAHO models had lower agreement. For many food categories, the FDA’s definition of “high-in” would require the fewest products to carry a “high-in” label. This issue was particularly pronounced in categories that tend to be served in small portions (e.g., salty snacks, bars), but disappeared or reversed for categories that are served in larger portions (e.g., frozen and non-frozen main dishes). **Conclusions**: The NPM chosen has important policy implications for an FOPL system’s ability to identify unhealthy foods and incentivize companies to reformulate products. Based on these results, the FDA should consider using a stronger NPM similar to those used elsewhere in the Americas region when deciding the final thresholds for “high-in” for US packaged foods and beverages.

## 1. Introduction

It is widely understood that the US food supply exposes Americans to food and beverage products that are high in saturated fat, sodium, and sugar (HFSS), and that these foods are linked to many chronic conditions. Packaged foods and beverages contribute ~75% of daily calorie intake in the US population [1], and at any point in time there are more than 400,000 unique products available on grocery store shelves. Deciding which of these many thousands of products constitute a healthy choice may not always be straightforward for US consumers. Front-of-package labels (FOPLs) on foods and beverages can help inform consumers when products are HFSS [2]. The US Biden–Harris Administration National Strategy on Hunger, Nutrition, and Health called for an FOPL system to make nutrition information more accessible to the US population [3]. In response, the US Food and Drug Administration (FDA) is in the process of testing and proposing an FOPL for foods and beverages high in nutrients of concern for chronic disease prevention [4], including sugar, sodium, and saturated fat, representing an important step towards helping consumers identify and reduce their consumption of these products. Such policies also have an important second benefit, which is to incentivize companies to reformulate products to contain less of the nutrients that are required to be labeled [5,6]. 

Developing an FOPL typically requires two steps: (1) testing the most effective visual design for communicating nutrition information to consumers and (2) deciding on a set of nutritional criteria for what should be considered “high in” a nutrient of concern. Most research on FOPLs focuses on the former, but the development of a nutrient profile model (NPM) is equally critical because it determines which foods receive a “high-in” FOPL. There are pros and cons to different NPM approaches. NPMs that do not use sufficiently rigorous nutrient thresholds are at risk of failing to identify all unhealthy foods in the food supply; they also may not stimulate companies to reformulate as many products since fewer products will be deemed as “high in”. Meanwhile, NPMs that are overly strict might result in such a high level of saturation of FOPLs on the food supply that they are less effective at guiding consumer choices or incentivizing reformulation. 

In addition, other issues relate to how the nutrient content is measured: for example, as percent of calories, percent of daily value, or per 100 g or 100 mL, all of which have implications for which products receive a “high-in” designation. Although the FDA has not yet published their proposed NPM which would underpin the FOPL, in their research they appear to use a threshold of ≥20% of the recommended daily value (%DV) per portion or serving size to define “high in” sodium, sugar, and saturated fat [4]. The ≥20% cutoff in the proposed FDA model aligns with “high source” claims for nutrient content claims [7]. The FDA’s definition for “high-in” as ≥20%DV was established in 1993 through the Nutrition Labeling and Education Act as part of FDA efforts to educate consumers and help promote healthier choices [8]. In recent years, other NPMs have been developed across the Americas, all with the purpose of informing policies to prevent diet-related non-communicable chronic diseases and some specifically designed for FOPL policies. All NPMs are set in reference to population-level guidance on nutrient intake. However, large differences exist in how the models are actually created. For example, the Pan American Health Organization (PAHO) NPM identifies foods and beverages as “excessive” in a nutrient of concern if its relative nutrient content (as a percent of calories) is higher than the corresponding maximum level in the World Health Organization Population Nutrient Intake Goals to Prevent Obesity and Related Non-Communicable Diseases [9]. The Chilean Government was the first country to implement mandatory FOPLs on “high-in” foods. Their NPM stipulates FOP warning labels for foods and drinks with high levels of added saturated fat, sodium, and sugar, with thresholds based on per 100 g or per 100 mL of product. Other governments have followed similar approaches to the PAHO and Chilean approach: for example, in 2020, the government of Mexico and the government of Brazil [10,11] both implemented mandatory FOPLs on products that are “high in” nutrients of concern with thresholds for Mexico based on the PAHO NPM (with slight modifications) and with thresholds for Brazil based on a per 100 g/mL approach. In addition, the government of Canada has enacted an FOPL which must appear by 2026 on products that are “high in” saturated fat, sugars, or sodium, with thresholds based on the %DV per portion [12], and with adjustments for the reference amount of a particular food, as established by Health Canada.

Understanding how the proposed FDA definition of “high-in” compares to established regional models is critical for informing future regulatory actions as well as understanding implications for the potential impact of the FOPL regulation in the US. The objective of this study was therefore to compare the proportion of products in the US food supply that would be considered “high-in” and thus receive an FOPL under the proposed US FDA approach to existing regional NPMs, including a modified version of Canada’s NPM for FOPLs, Chile’s NPM, and the PAHO NPM. Due to the Mexico and Brazil models being heavily based on other existing NPMs, these were not examined in analysis.

## 2. Materials and Methods

This study was not human subjects research and thus did not require Institutional Review Board approval. The data used in this study are proprietary and available via institutional contract with Mintel. The statistical code used to generate the results can be found in the Open Science Framework at DOI 10.17605/OSF.IO/PC4FX.

Mintel data: Mintel’s Global New Products Database (GNPD) maintains a global, web-based database of newly launched consumer packaged goods in key global markets (“newly launched” includes any products that were introduced, had formulations change, or included any changes to the packaging) [13]. Mintel secret shoppers photograph products and enter package information manually into the Mintel system. The Global Food Research Program (GFRP) utilizes Nutrition Facts Label (NFL) data from the Mintel GNPD for food and beverage products. GFRP programmers download product information from the Mintel website to create the GFRP Mintel NFL database. This study includes packaged food and beverage products from Mintel 2019–2023 collected in the USA. Mintel records from the North American region were downloaded and processed (*n* = 112,376). Records were excluded if ingredient lists were missing (*n* = 2555); for single-ingredient products (*n* = 9077); for non-ready to drink beverages (*n* = 791; e.g., beverage concentrates/mixes, coffee, tea); for categories not of interest or requiring preparation (*n* = 11,016; e.g., alcoholic beverages, baby food, baking mixes, nutritional supplements and meal replacements, culinary ingredients); if nutrient values for added sugar, sodium, or saturated fat were missing (*n* = 22,205); for products collected in Canada (*n* = 9444); for duplicate barcodes (*n* = 5199); and plain milk products exempt from some FOPL systems (*n* = 280). The final sample included 51,809 products (47,503 food and 4306 beverages).

Food grouping system: Mintel GNPD includes data from food and beverage products in over 120 subcategories. These subcategories were combined into food groupings as presented in Table S1 for foods and Table S2 for beverages.

Nutrient profile models (NPMs): Each NPM uses a different set of thresholds and process for determining when a product is “high in” sodium, sugar (either added or total sugar), or saturated fat. This can affect how many products would potentially receive a “high-in” label under different NPMs. Although NPMs also vary in which nutrients can be considered “high-in” or be eligible for a warning label (e.g., the Chilean model includes calories and PAHO includes trans fat), this study focused only on the nutrients under consideration by the FDA: sugar, sodium, and saturated fat.

NPM for FDA FOPL research: The FDA has not issued any final rule, but the NPM currently utilized in the FDA’s ongoing FOPL research includes thresholds based on %DVs of saturated fat, sodium, or added sugars in prepackaged products [4]. The designation of “high-in” depends on whether a nutrient value in a portion exceeds the 20% threshold for the contribution of that nutrient to the total daily recommended consumption of that nutrient. The thresholds are the same for each nutrient, serving size, and category of product: High ≥ 20%DV.

NPM for Canada’s FOPL system: The Canadian NPM thresholds used for their forthcoming FOPL system are based on %DVs of saturated fat, sodium, or added sugars in prepackaged products [12]. The thresholds for “high-in” labels are dependent on “reference amount”/serving size: ≥10%DV for products with reference amount 30 g or 30 mL or smaller, ≥15%DV for products with reference amount over 30 g or 30 mL, and ≥30%DV for main dishes.

While the official method of calculation for the Canadian NPM involves adjusting nutrients to align serving sizes with reference amounts for each product category, our analysis utilized a simplified approach, applying ≥10% for serving sizes of 30 g or 30 mL or smaller, ≥15% for serving sizes over 30 g or 30 mL, and ≥30% for main dishes with serving sizes over 200 g.

NPM for Chile’s FOPL system: The Chilean NPM included staggered nutrient thresholds enacted in 2016, 2018, and 2019 [14]. For this analysis, the final thresholds were applied. Solid foods (sold in grams) with added fat, sugar, or sodium ingredients are regulated when 100 g of product contains >4 g saturated fats, >10 g sugar, or >400 mg sodium. Beverages or liquid foods (sold in mL) with added fat, total sugar, or sodium ingredients are regulated when 100 mL of product contains >3 g saturated fats, >5 g sugar, or >100 mg sodium.

Pan American Health Organization (PAHO) NPM: The PAHO NPM is applied to processed and ultra-processed foods [9]. For this research, packaged products that included added ingredients for sugar, sodium, or fat ingredients were considered as processed or ultra-processed and eligible for application of PAHO thresholds. The PAHO NPM uses calories as the denominator for determining “high-in” thresholds. A product is considered “high-in” according to the PAHO NPM if it has ≥1 mg sodium per 1 calorie, ≥10% of total energy from free sugars, or ≥10% of total energy from saturated fat. Although the PAHO NPM uses free sugars (which includes all sugars from fruits/vegetables, including fruit juices, concentrates, or dried fruit), due to labeling requirements in the US, we used added sugar (which includes fruit juice concentrates over the amount found in 100% juice but not other sugars from fruit/vegetables) to align with US FDA labeling requirements.

Statistical analysis: Data were analyzed using SAS 9.4. The number and proportion of products defined as “high-in” under each NPM was calculated overall and by food and beverage category. Products were flagged as either concordant when considering all 4 NPMs (e.g., a product is considered “high-in” under all 4 NPMs, or considered not being “high-in” under all 4 NPMs), or as discordant (e.g., a product is not considered “high-in” under one NPM but is considered “high-in” under a second NPM). The percent agreement among the 4 NPMs was calculated as the percent of concordant products out of the total number of products. Fleiss’ kappa statistic was used to explore agreement between the proportion of products considered being “high-in” comparing the FDA ≥ 20% DV NPM with each of the other 3 NPMs.

## 3. Results

### 3.1. Proportion of Products That Met the “High-In” Criteria Under Each NPM, Overall

**Figure 1:**
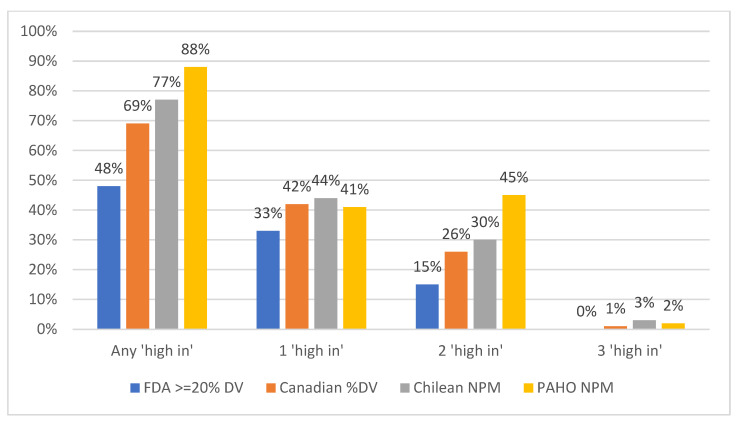
Percent of US food products that would be considered ‘high in’ added sugar, saturated fat, and/or sodium among four nutrient profile models (NPMs) based on % Daily Value (DV), Chile, or the Pan American Health Organization (PAHO) using data from Mintel USA (2019–2023; *n* = 47,503 food products). “Any ‘high in’” refers to products that meet criteria for one or more nutrients (i.e., added sugar, saturated fat, sodium); “1 ‘high in’”, “2 ‘high in’”, or “3 ‘high in’” refers to the number of nutrients a product meets criteria for high in added sugar, saturated fat, and/or sodium.

**Figure 2:**
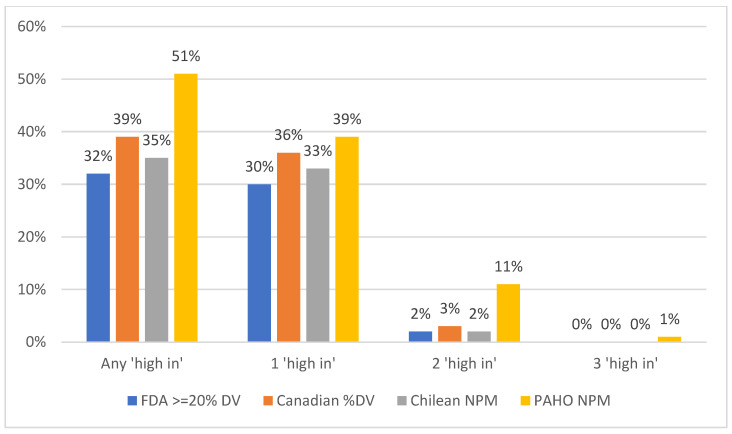
Percent of US beverage products that would be considered ‘high in’ added sugar, saturated fat, and/or sodium among four nutrient profile models (NPMs) based on % Daily Value (DV), Chile, or the Pan American Health Organization (PAHO) using data from Mintel USA (2019–2023; *n* = 4306 beverage products). “Any ‘high in’” refers to products that meet criteria for one or more nutrients (i.e., added sugar, saturated fat, sodium); “1 ‘high in’”, “2 ‘high in’”, or “3 ‘high in’” refers to the number of nutrients a product meets criteria for high in added sugar, saturated fat, and/or sodium.

Under the NPM proposed by the US FDA, about half of the 47,503 foods examined (48%) and a third of the 4306 beverages (32%) could be expected to bear at least one “high-in” warning label by meeting criteria for high in added sugar, saturated fat, and/or sodium (Figure 1 and Figure 2). The FDA’s proposed NPM was less strict than the three existing regional NPMs and the PAHO NPM the strictest, with 88% of foods and 51% of beverages expected to bear at least one “high-in” warning label. The Canadian NPM was the least strict after the US FDA NPM for foods, but not for beverages, with the Chilean NPM the least strict NPM for beverages after the FDA NPM. The Chilean NPM classified a similar proportion of beverage products (35%) as “high-in” to the FDA NPM (32%).

### 3.2. Proportion of Products That Met the “High-In” Criteria Under Each NPM, by Food Category

Overall, there was 51% agreement for food products between all four NPMs examined. Category-specific results (Table 1) confirm that the PAHO NPM would result in the greatest coverage of food products (i.e., would result in the most “high-in” warning labels), with the FDA’s proposed NPM the least. Agreement overall was highest between the FDA and the Canadian NPM, with six categories showing substantial agreement (i.e., k ≥ 0.61). The PAHO NPM showed the lowest agreement with the FDA’s proposed NPM, with more than half of all food categories receiving only slight agreement (k = 0.01–0.2).

The FDA’s proposed NPM was the least strict of the four NPMs examined for 14 of the 17 food categories. For example, the FDA’s proposed NPM resulted in only 22% of ‘salty snacks’ products receiving a “high-in” warning label, far lower than the Canadian NPM (59%), PAHO NPM (84%), and Chilean NPM (89%). Agreement was higher for ‘sweet snacks’ (86%) and ‘desserts’ (76%). Other notable examples were that only 10% and 25% of ‘bread products’ would bear a “high-in” label under the FDA and Canadian NPMs, soaring to 78% and 95% under the Chilean and PAHO NPMs. ‘Bread products’ was in fact the category with the lowest agreement overall between the four NPMs (14%). Under the PAHO NPM (the strictest NPM), the only food groups in which less than 75% of products would bear at least one “high-in” warning label were ‘fruits and vegetables’ and ‘side dishes’; the rest of the categories had a very high proportion of products (≥75%) that would be expected to bear at least one “high-in” warning label. Conversely, only ‘soup’, ‘sweet snacks’, and ‘frozen meals’ exceeded 75% of products receiving at least one “high-in” warning label under the FDA’s proposed NPM.

### 3.3. Proportion of Products That Met the “High-In” Criteria Under Each NPM, by Beverage Category

For beverage categories, the proportion of products that would receive at least one “high-in” warning label under each NPM was not as wide-ranging as food categories—from 32% under the FDA NPM to 51% under the PAHO NPM (Table 2). Overall agreement between the FDA’s proposed NPM and the Canadian NPM was high (k = 0.84), with substantial agreement (k ≥ 0.81) in six of the 10 beverage categories examined. Only one beverage category (‘juice’) showed substantial agreement between the FDA’s proposed NPM and the PAHO NPM and the Chilean NPM. All of the NPMs analyzed resulted in a vast majority (83–93%) of ‘non-diet soda’ but only a select few (<10%) of ‘flavored and regular waters’ receiving at least one “high-in” warning label. The beverage categories with the greatest disparities were dairy drinks. Interestingly, 83% of ‘non-plain dairy drinks’ and ‘flavored milk’ would receive at least one “high-in” warning label under the PAHO NPM, while less than half of products in this category would receive a “high-in” warning label under the other three NPMs.

### 3.4. Proportion of Products That Met Each “High-In” Warning Labels’ Criteria Under the FDA’s Proposed NPM

Of the three nutrients subject to “high-in” warning labeling, the most common for foods under the FDA’s proposed NPM would be for saturated fat, with 27% of products receiving a “high in” saturated fat warning label, followed by added sugar (22%) then sodium (15%; Table 3). ‘Cheese, butter, and other dairy’ would receive the highest proportion of “high in” saturated fat warning labels (71%) followed by ‘main dish—frozen’ (65%). Less than 5% of ‘bread products’, ‘fruit and vegetables’, and ‘sauces, dips, and seasoning’ would receive a “high in” saturated fat warning label. ‘Sweet snacks’ would receive the highest proportion of “high in” added sugar warning labels (67%) followed by ‘desserts’ (62%) and ‘breakfast cereals’ (48%). Less than 5% of ‘side dishes’, ‘cheese, butter, and other dairy’, ‘proteins’, ‘bread products’, and ‘sauces, dips, and seasonings’ would receive a “high in” added sugar warning label under the FDA’s proposed NPM. Greater than or equal to two thirds of ‘main dish—frozen’, ‘main dish—non-frozen’, and ‘soup’ would receive a “high in” sodium warning label, with 11 of the 19 included categories having <10% products receiving a “high in” sodium warning label. Less than 10% of ‘salty snacks’, for example, would receive a “high in” sodium warning label under the FDA’s proposed NPM.

For beverages, 30% of products overall would receive a “high in” added sugar warning label under the FDA’s proposed NPM (Table 4). Only 3% would receive a “high in” saturated fat warning label and only 1% would receive a “high in” sodium warning label. Not surprisingly, ‘regular sodas’ was the category that would receive the highest proportion of “high in” added sugar warning labels (84%), followed by ‘ready-to-drink coffee and tea’ (50%). ‘Diet sodas’ and ‘water’ were the only categories with zero products receiving any “high-in” warning labels under the FDA’s proposed NPM.

## 4. Discussion

If the FDA’s proposed ≥20% DV criteria were applied to US packaged foods and beverages and companies did not reformulate, 48% of foods and 32% of beverages would receive at least one “high-in” warning label. Although all NPMs have pros and cons, the FDA’s proposed “high-in” criteria were substantially less strict compared to the Canadian NPM, Chilean NPM, and PAHO NPM. Similarly, only 15% of foods would have two “high-in” warning labels under the US FDA approach, versus between 26% and 45% under other regional NPMs. The lower prevalence of “high-in” labels under the US FDA model raises questions as to whether the use of ≥20% DV as the threshold for “high-in” labels may underestimate and thus under-identify foods and beverages high in sodium, saturated fat, and added sugar in the US food supply. Such an underestimation could limit the public health benefit of an FOPL system in terms of helping consumers identify unhealthy foods and incentivizing companies to reformulate them.

Many of the differences observed in this study between the FDA’s proposed NPM and the three established regional NPMs can be explained by a number of key differences in the underlying algorithms used to define “high-in”. For example, the Chilean model uses the presence of an added sugar ingredient and total sugar values to assign sugar thresholds, while the other models all used added sugar. However, perhaps the biggest difference is the denominator with which the nutrient thresholds are derived. The FDA and Canadian thresholds are based on the nutrition content per serving (%DV), likely due to precedent: nutrition information per serving (rather than per 100 g or mL) is what is already required on the back of the package. On the other hand, the Chilean NPM uses nutrient criteria per 100 g/mL when defining “high-in” and the PAHO NPM uses density (percent or a ratio of the nutrient to total calories). These differences mean that for the US FDA and Canada, nutrient thresholds are relative for a particular portion’s contributions to the total daily diet (i.e., smaller portions will have smaller %DV) whereas the nutrient thresholds for Chile and PAHO are constant regardless of the portion. However, a key difference between the US and Canada is that the FDA model uses ≥20% for all products, regardless of reference amount. In contrast, the Canadian approach establishes stricter thresholds for products with smaller reference amounts, reflecting that these products contribute overall less to the daily diet and thus should also contribute less to the total daily intake of added sugar, sodium, and saturated fat. For example, in Canada, a 24 g granola bar would only need to contain 5 g of added sugar (10% of DV) to receive a “high in” sugar label, whereas a main dish would have to contain 15 g of added sugar (30% of DV) to receive the label.

The differences between NPMs were apparent in the overall low agreement between the four NPMs (51%) for packaged food products as well as the heterogeneity by category. Generally, agreement was lowest for savory-type food categories such as salty snacks and bread. The US FDA NPM was the most lax in these categories compared to the remaining three NPMs, mostly due to differences in sodium thresholds. For example, bread products tend to receive a “high in” sodium label under the PAHO and the Chilean NPMs. The PAHO “high in” sodium criteria are based on a >1:1 sodium to calorie ratio, which many bread products exceed even while providing relatively small amounts of both sodium and calories within a single serving. The Chilean “high in” sodium criteria are based on a per 100 g denominator; therefore, food products which are lighter in weight can exceed the limit of 400 mg/100 g sodium. Using the %DV approaches, a serving of two slices of bread would provide sodium amounts that would not exceed either the FDA or Canadian NPMs (example: 50 g serving of bread with 210 mg sodium is 9%DV in the US). Only 15% of food products would receive a “high in” sodium warning label under the FDA’s proposed criteria. This suggests that the FDA’s 20% DV approach may miss a sizeable proportion of high-sodium-density products in the US. As research has shown that US sodium intake far exceeds recommended levels [15] and is mostly attributed to store-bought packaged foods [16,17], these results suggest that the 20%DV sodium threshold may be too low for the US food supply. Given the FDA has previously released draft sodium reduction targets [18], it would seem fair to suggest that the proposed approach using ≥20% DV for sodium would not be appropriate for application across the US packaged food supply.

The huge discrepancies seen for salty snacks were mainly due to many products being sold in small portions (pack sizes). Because their serving sizes may be small, salty snacks are unlikely to contribute a high %DV even though their nutrient density is high. Even though these foods are purportedly served in small portions, many snack foods are ultraprocessed, designed to be hyperpalatable, and are easily overconsumed. This raises questions as to the utility of the %DV threshold as an appropriate approach for an FOPL in the US with its very high prevalence of snacking (i.e., according to the National Health and Nutrition Survey, 95% of adults consume at least one snack a day) [19]. For example, a large package of Cheetos (17.5 oz) specifies a serving size of 28 g (21 pieces, with 17 servings per package). Under the proposed FDA %DV approach, no “high-in” labels would be displayed. However, under the modified Canadian NPM, which adapts the %DV approach for portion size, “high in” sodium labels would be displayed, and under the Chilean NPM both “high in” sodium and saturated fat labels would be displayed. It is highly unlikely that a consumer would eat the contents of the 17.5 oz Cheetos pack in 17 different sittings and highlights the unsuitability of using portion sizes as the underlying measure for an NPM underpinning FOPL policies in the US.

Other categories with large discrepancies were also likely because they are typically served in small portions: for example, only 18% of sauces would have a “high-in” label under the FDA %DV approach versus 49% to 89% under other regional profile models, with similar results for sweet spreads and syrups. Breakfast-related items also showed large discrepancies: for cold cereals, the FDA %DV approach identified 56% as “high-in” compared to 77% to 91% under other NPMs and for bars, the FDA %DV approach identified 39% as “high-in” compared to 70% to 88% under other NPMs.

Ultimately, these results show that the US’s %DV approach is less well-suited to identify products that have a high density of added sugar, sodium, or saturated fat if the portion that is consumed is small. This is problematic for several reasons: first, it could lead to consumer confusion if products that have larger portions are flagged as “high-in” but products with small portions but high density of nutrients are not. Second, although US portion sizes must align with the FDA-determined Reference Amount Customarily Consumed (RACC) and be reported in a common household measure appropriate to the food, manufacturers could reduce portion sizes to avoid labels if close to thresholds. Perhaps more importantly, an ample and growing body of research shows that ultra-processed and hyperpalatable foods high in sugar, sodium, and saturated fat have addictive properties that promote overconsumption [20]. A label that is relative to portion size is less likely to be helpful if consumers are eating multiple portions. Third, the %DV is in reference to an adult’s daily diet, limiting its utility on toddler and children’s snacks.

It is worth noting that all NPMs have pros and cons, and there were some areas where the US FDA approach was similar to other regional NPMs or performed better. For example, the US FDA approach was similar to or performed better than other regional NPM categories for some foods (sweet snacks, soups, yogurts, main dishes). For some groups, like main dishes, the higher prevalence of HFSS identified by the US FDA approach is again likely due to portion size, since these dishes are intended to represent a larger proportion of daily diets. For other categories, like sweet snacks, these tend to contain such high amounts of sugar that they are likely to exceed the %DV threshold even with small portions. Sweet snacks products also tend to have heavier weights, so there isn’t the issue of a lightweight product faring differently with a weight-based denominator vs a calorie/DV denominator (in contrast to many salty snacks like chips). In addition, there was high agreement for beverages overall and in most subcategories (e.g., between 84% and 93% of sodas were identified as “high-in”). The higher agreement for beverages is likely because added sugar is the most likely nutrient to be “high-in” in beverages and most sugary drinks exceed the nutrient thresholds across NPMs, though there were still discrepancies related to portion size (e.g., other dairy liquid, which is comprised mainly of flavored creamers, had only 5% “high-in” under the US FDA compared to 58% to 71% under other approaches). In addition, there is concern about overly restrictive FOPLs. For example, under the PAHO model, 88% of foods would have at least one “high-in” label. Such a high coverage of labels on foods may dilute the utility of labels in informing choice, since consumers would have few options without a “high-in” label. 

In addition to influencing consumer choice, the NPM can also influence the extent to which an FOPL system incentivizes companies to reduce nutrients of concern across their portfolios. Such reformulation is an important additional benefit, given that the US packaged food and beverage supply provides the majority of dietary sodium, saturated fat, and added sugars to the average American diet. Indeed, previous studies have shown that FOPLs encourage the food industry to reformulate their products [21,22]. Research from countries like Chile and Peru, which were among the first to implement mandated “high-in” FOPL systems (based on per 100 g/mL nutrient criteria), show that reductions usually occur right around the nutrient thresholds established by the NPM [23,24]. Thus, the choice of NPM has implications for how a given FOPL system works for reducing the content of nutrients of concern in the food supply. NPMs that are too strict may not stimulate reformulation since it may not be achievable for manufacturers, whereas NPMs that are too lax may not stimulate reformulation since not as many products will be subject to receive a label.

This study has important policy implications. First, it is important to recognize that in the US, adoption of a strong NPM likely faces several external barriers, including legal barriers, the need to rely on previous precedent (e.g., %DV), and, as in many countries, broader food industry interference in the policymaking agenda (e.g., through lobbying politicians who can delay or circumvent regulatory action) [25,26,27,28]. However, the US FDA could still strengthen their NPM by adopting an approach similar to Canada’s, which provides adjusted thresholds for foods offered in smaller portions. Moreover, the US could follow approaches used by many other countries, which is to strengthen the NPM over time, giving the food industry more time to adapt and reformulate products. Together, these approaches would allow the US to implement an NPM that adequately identifies foods high in nutrients of concern, informs consumers, and stimulates a healthier food supply.

This study is not without limitations. Mintel’s GNPD only holds data for products that are new to the market (or that have been reformulated and/or have new packaging). Due to this, it is possible that products that have remained on the US market for many years were not captured in this analysis. In addition, some food categories had relatively low sample sizes and may not be representative of the market for that category. Lastly, this dataset reflects products, not necessarily those that are most consumed. To better understand the health implications of different NPMs, future research should use large, nationally representative datasets of food purchases and dietary intake.

Another key consideration is that the FDA’s proposed NPM does not take into account the level of processing a product has undergone, and as such was not examined in this study. There is a wealth of research over the past decade showing the ultra-processed foods that now dominate US diets, with one recent study even showing that HFSS-based approaches (similar to the four NPMs examined in this study) often “miss” ultra-processed foods (and vice versa) and may help ensure that US policymakers have both a simple and accurate method for the identification of less healthy food and beverage products [29].

## 5. Conclusions

With packaged foods contributing more than two thirds of the diet of the average American, the implementation of FOPLs on US foods and beverages would undoubtedly be a useful tool for many consumers trying to improve the healthfulness of their diets. NPMs are critical for determining the coverage of FOPLs in the food supply and influencing how the FOPL system informs consumers and stimulates reformulation. This study found that the FDA’s likely NPM of ≥20% DV would result in a lower proportion of products carrying a “high-in” label relative to other established regional NPMs, particularly for foods, which could limit a subsequent FOPL system’s public health benefit. To address this, the US should consider implementing different %DV thresholds that account for different reference portion sizes, as is done in Canada, and implementing stronger thresholds over time. Additional research using national data on food purchases could additionally help understand how different NPM approaches would affect the foods and beverages most commonly consumed by Americans and the subsequent implications for chronic disease prevention.

## Figures and Tables

**Table 1:** Percent of US food products that would be considered ‘high-in’ comparing nutrient profile models (NPMs) of added sugar, saturated fat, and sodium based on % Daily Value (DV), Chile, or PAHO using data from Mintel USA (2019–2023; *n* = 47,503 food products).

Food Group	*n*	FDA ≥ 20% DV	Canadian %DV	Chilean NPM	PAHO NPM	% Agreement Between All 4 NPMs	Kappa Canadian %DV	Kappa Chilean NPM	Kappa PAHO NPM
All food products	47,503	48	69	77	88	51	0.56	0.22	0.20
Bars	1506	39	70	90	88	45	0.43	0.10	0.14
Breakfast cereals	1377	56	77	91	91	61	0.55	0.18	0.21
Hot cereals	289	42	62	72	80	52	0.61	0.18	0.30
Bread products	1493	10	25	78	95	14	0.49	0.05	0.01
Cheese, butter, and other dairy	3312	72	92	85	96	63	0.35	0.09	0.02
Desserts	6025	74	92	96	97	76	0.41	0.19	0.14
Main dish—frozen	1589	92	85	56	99	55	0.32	0.13	0.20
Main dish—non-frozen	1582	71	79	55	97	50	0.55	0.38	0.08
Fruit and vegetables	1863	13	24	13	63	45	0.66	0.54	0.16
Proteins	4449	56	79	72	92	55	0.50	0.40	0.12
Side dishes	2267	29	42	29	52	68	0.72	0.61	0.52
Salty snacks	7260	22	59	89	84	29	0.33	0.05	0.10
Sweet snacks	5571	77	87	89	89	86	0.65	0.53	0.54
Sauces, dips, and seasonings	6313	18	49	81	89	20	0.38	0.02	0.03
Sweet spreads and syrups	1173	31	71	86	83	39	0.31	0.10	0.17
Soup	640	79	86	33	96	32	0.74	0.09	0.24
Yogurt	794	53	66	32	74	49	0.74	0.52	0.32

FDA = Food and Drug Administration; DV = Daily Value; NPM = nutrient profile model; PAHO = Pan American Health Organization. % agreement = product meets criteria for all four NPMs as either no FOPs or 1 or more FOPs. Kappa values are comparisons with FDA ≥ 20% DV.

**Table 2:** Percent of US beverage products that would be considered ‘high-in’ comparing nutrient profile models (NPMs) of added sugar, saturated fat, and sodium based on % Daily Value (DV), Chile, or PAHO using data from Mintel USA (2019–2023; *n* = 4306 beverage products).

Beverage Group	*n*	FDA ≥ 20% DV	Canadian %DV	Chilean NPM	PAHO NPM	% Agreement Between All 4 NPMs	Kappa Canadian %DV	Kappa Chilean NPM	Kappa PAHO NPM
All beverage products	4306	32	39	35	51	72	0.84	0.68	0.59
Dairy drinks and flavored milks	511	35	46	41	83	36	0.77	0.43	0.06
Other dairy liquid	381	9	58	69	71	18	0.13	0.09	0.08
Fermented drinks	83	39	48	8	65	40	0.81	0.08	0.50
Flavored water	681	1	1	0	6	94	0.93		0.24
Juice and juice drinks	915	35	37	34	43	88	0.96	0.87	0.83
Ready-to-drink coffee and tea	509	51	55	41	65	74	0.93	0.69	0.73
Sodas—regular	481	84	87	83	93	87	0.91	0.77	0.56
Sodas—diet	244	0	0	0	18	82			
Sports and energy drinks	412	33	38	23	50	69	0.89	0.75	0.61
Water	89	0	0	0	0	100			

FDA = Food and Drug Administration; DV = Daily Value; NPM = nutrient profile model; PAHO = Pan American Health Organization. % agreement = product meets criteria for all four NPMs as either no FOPs or 1 or more FOPs. Kappa values are comparisons with FDA ≥ 20% DV.

**Table 3:** Percent of US food products that would be considered ‘high in’ added sugar, sodium, and saturated fat based on 20% Daily Value (DV) using data from Mintel USA (2019–2023; *n* = 47,503 food products).

Food Group	*n*	FDA ≥ 20% DV Added Sugar	FDA ≥ 20% DV Sodium	FDA ≥ 20% DV Saturated Fat
All food products	47,503	22	15	27
Bars	1506	19	0	24
Breakfast cereals	1377	48	0	10
Hot cereals	289	35	2	7
Bread products	1493	1	6	4
Cheese, butter, and other dairy	3312	0	2	71
Desserts	6025	62	1	49
Main dish—frozen	1589	5	85	65
Main dish—non-frozen	1582	6	65	32
Fruit and vegetables	1863	5	8	2
Proteins	4449	2	45	25
Side dishes	2267	0	24	9
Salty snacks	7260	6	7	14
Sweet snacks	5571	67	0	42
Sauces, dips, and seasonings	6313	4	12	4
Sweet spreads and syrups	1173	24	0	9
Soup	640	2	77	20
Yogurt	794	37	0	28

FDA = Food and Drug Administration; DV = Daily Value.

**Table 4:** Percent of US beverage products that would be considered ‘high in’ added sugar, sodium, and saturated fat based on 20% Daily Value (DV), using data from Mintel USA (2019–2023; *n* = 4306 beverage products).

Beverage Group *	*n*	FDA ≥ 20% DV Added Sugar	FDA ≥ 20% DV Sodium	FDA ≥ 20% DV Saturated Fat
All beverage products	4306	30	1	3
Dairy drinks and flavored milks	511	25	0	14
Other dairy liquid	381	7	0	7
Fermented drinks	83	37	0	1
Flavored water	681	1	0	0
Juice and juice drinks	915	34	2	0
Ready-to-drink coffee and tea	509	50	0	4
Sodas—regular	481	84	1	0
Sodas—diet	244	0	0	0
Sports and energy drinks	412	30	5	0
Water	89	0	0	0

FDA = Food and Drug Administration; DV = Daily Value; * plain milks excluded from analysis due to exclusion from some NPMs.

## Data Availability

These data are proprietary secondary data and the authors do not have legal permission to share them. Readers interested in using these data should contact Alison Agnew at aagnew@mintel.com.

## Supplementary Materials

The following supporting information can be downloaded at https://www.mdpi.com/article/10.3390/nu16244345/s1, Table S1: Food groupings created based on Mintel GNPD subcategories with examples of products included in each food group; Table S2: Beverage groupings created based on Mintel GNPD subcategories with examples of products included in each beverage group.
