# Supplementary material for: Defining “High-In” Saturated Fat, Sugar, and Sodium to Help Inform Front-of-Pack Labeling Efforts for Packaged Foods and Beverages in the United States
Source: Nutrients. 2024 Dec 17;16(24):4345. doi: 10.3390/nu16244345 (PMC11677797; doi:10.3390/nu16244345)
Supplement: Supplementary file 1 [file nutrients-16-04345-s001.zip › nutrients-3326981-supplementary.pdf]

Supplementary Tables for: ***Defining "High-In" Saturated Fat, Sugar, and Sodium to Help Inform Front-of-Pack Labeling Efforts for Packaged Foods and Beverages in the United States.***

Table S1. Food groupings created based on Mintel GNPD subcategories with examples of products included in each food group.

Food Group Name	Description of products included in Food Group
Bars	Cereal Bars, Granola Bars, Snack Bars, Energy Bars
Bread Products	Bread, Rolls, Bread Products
Breakfast Cereals	Ready-to-Eat / Cold Breakfast Cereals
Cheese, Butter, & Other Dairy	Hard Cheese, Soft Cheese, Cream Cheese, Processed Cheese, Curd, Quark, Sour Cream, Butter, Margarine
Desserts	Cakes, Pastries, Cookies, Ice Cream, Cheesecake, Frozen Desserts
Main Dish - Frozen	Pizza, Pasta Dishes, Rice Dishes, Sandwiches
Main Dish - Non-Frozen	Salads, Pasta Dishes, Rice Dishes, Sandwiches, Wraps
Fruit & Vegetables	Packaged Fruits & Vegetables, Fresh, Frozen, Canned, or Dried
Hot Cereals	Hot Breakfast Cereals, Oatmeal, Cream of Wheat/Rice
Proteins	Meat, Fish, Poultry, Eggs
Salty Snacks	Chips, Crackers, Popcorn, Nuts, Snack Mixes, Jerky
Sauces, Dips, & Seasonings	Salad Dressing, Mayonnaise, Ketchup, Mustard, Barbecue Sauce, Pasta Sauce, Soy Sauce, other Table Sauces, Dips, Spreads
Side Dishes	Potato Products, Stuffing, Flavored Rice Mixes, Pasta, Noodles
Soup	Soup
Sweet Snacks	Fruit Snacks, Candies, Chocolates
Sweet Spreads & Syrups	Peanut Butter, Jams, Preserves, Jelly, Syrup
Yogurt	Yogurt

Table S2. Beverage groupings created based on Mintel GNPD subcategories with examples of products included in each beverage group.

Beverage Group Name	Description of products included in Beverage Group
Coffee & Tea	Ready-to-Drink / Iced Coffee & Tea
Dairy Drinks & Flavored Milks	Flavored Milks, Dairy-based Beverages, Liquid Yogurt, Plant-based Milk Alternatives
Fermented Drinks	Kombucha, other Non-alcoholic Fermented Beverages
Flavored Water	Flavored Waters
Juice & Juice Drinks	Juice, Nectars, Juice Drinks
Other Dairy Liquid	Cream, Creamers
Sodas - Diet	Carbonated Soft Drinks with <10kcal/100mL
Sodas - Regular	Carbonated Soft Drinks with ≥10kcal/100mL
Sports & Energy Drinks	Sports Drinks, Energy Drinks
Water	Plain Water
